# Definitive radio(chemo)therapy *versus* upfront surgery in the treatment of HPV-related localized or locally advanced oropharyngeal squamous cell carcinoma

**DOI:** 10.1371/journal.pone.0307658

**Published:** 2024-07-25

**Authors:** Jérémy Baude, Caroline Guigou, David Thibouw, Noémie Vulquin, Mireille Folia, Guillaume Constantin, Jihane Boustani, Christian Duvillard, Sylvain Ladoire, Gilles Truc, Aurélie Bertaut, Cédric Chevalier

**Affiliations:** 1 Department of Radiotherapy, Georges-François Leclerc Cancer Center, UNICANCER, Dijon, Burgundy, France; 2 Department of Otolaryngology and Head and Neck Surgery, Dijon University Hospital, Dijon, Burgundy, France; 3 Department of Radiation Oncology, Besançon University Hospital, Besançon, Burgundy, France; 4 Department of Epidemiology and Biostatistics, Georges-François Leclerc Cancer Center, Dijon, Burgundy, France; 5 Department of Medical Oncology, Georges-François Leclerc Cancer Center, Dijon, Burgundy, France; 6 Cancer Biology Transfer Platform, Georges-François Leclerc Cancer Center, Dijon, Burgundy, France; 7 INSERM LNC-UMR1231, Dijon, France; 8 Genetic and Immunology Medical Institute, Dijon, Burgundy, France; University of Vermont College of Medicine: University of Vermont Larner College of Medicine, UNITED STATES OF AMERICA

## Abstract

**Background:**

The treatment of stage I-III HPV+ oropharyngeal squamous cell carcinoma (HPV-OPSCC) is based on either surgery ± adjuvant therapy or exclusive radio±chemotherapy. We sought to compare these two therapeutic strategies in terms of efficacy, tolerance and quality of life (QoL).

**Methods:**

Patients treated for stage I-III HPV-OPSCC from 2010 to 2021 in 3 academic centers were included and sorted according to the treatment strategy: surgery or exclusive radio±chemotherapy. Efficacy and tolerance were retrospectively assessed, and a transversal exploratory QoL assessment was performed using QoL instruments.

**Results:**

A total of 83 patients were included, with 21 undergoing non-minimally invasive surgery and 62 receiving definitive radio-±chemotherapy. 2-year progression-free survival (PFS) and overall survival (OS) were respectively 80% and 86% in the surgical group and 92% and 95% in the non-surgical group, with no significant difference. At the end of treatment, 64.5% of patients presented with a grade III toxicity, without significant difference between the two groups. No patient had late grade III toxicity at 24 months. Forty-five patients (11 in the surgical group, 34 in the non-surgical group) participated in an exploratory quality-of-life analysis. Patients reported significantly more fatigue and loss of appetite after surgery, whereas patients in the radio±chemotherapy group described significantly more salivary and oral problems and difficulty swallowing, but the median time between treatment completion and the response to the questionnaires.

**Conclusion:**

There was no significant difference in efficacy, physician-reported toxicity and overall patient-reported quality of life was found between non-minimally invasive surgery and radio±chemotherapy in the treatment of stage I-III HPV-OPSCC.

## Background

The incidence of oropharyngeal squamous cell carcinoma (OPSCC) has substantially increased over the past twenty years and is likely to continue to rise [1]. The International Agency for Research on Cancer (IARC) projects that between 2020 and 2040, the incidence of OPSCC will increase by 8% in Europe and up to 15% in North America [2]. Mainly due to alcohol consumption and smoking in the past decades, it is now well established that a growing share of OPSCCs is attributable to human papillomavirus (HPV) infection [3]: 30-70% of OPSCCs in Europe and North America are now HPV-related [4].

HPV-related OPSCC (HPV-OPSCC) differs from non-HPV-related OPSCC, as they each present different molecular, histological and epidemiological characteristics [1]. Furthermore, It has been shown that HPV infection is an independent prognosis factor regardless of the chosen treatment [5, 6].

To date, no treatment has been proven to be superior to another in the management of localized and locally advanced OPSCCs. Irrespective of the HPV status, the treatment is based on either upfront surgery (uS), followed or not by adjuvant therapies – including radiation therapy (RT) and chemotherapy (CT) – or exclusive radiotherapy (eRT) or radio-chemotherapy (eRCT) [7, 8]. The only trial comparing these two strategies in stage III/IV OPSCCs had to be discontinued because it failed to reach the accrual goal [9] and included 119 patients with head and neck cancer, of whom only 42 patients had OPSCC. In less advanced diseases (T1-2 N0-2), the phase II ORATOR study compared transoral robotic surgery (TORS) with or without adjuvant treatments to eRT±CT in terms of quality of life but was not designed to assess the efficacy of both strategies [10].

The majority of data on the two options for the treatment of HPV-OPSCC are from monocentric studies [11, 12] or the American National Cancer Database [13]. Furthermore, since patients with HPV-OPSCC are younger, healthier and live longer, the impact of treatments on quality of life (QoL) is crucial [14]. However, apart from the ORATOR trial, the aforementioned studies did not focus on tolerance [11–13, 15], and data remain sparse.

Consequently, the objective of this multicentric work was to provide additional data concerning the efficacy and tolerance of surgical and non-surgical strategies in patients with localized or locally advanced HPV-OPSCC.

## Methods

### Patients

This retrospective study was conducted at three French tertiary referral centers in the management of OPSCCs: the Georges-François Leclerc Cancer Center (Dijon), the François Mitterrand University Hospital (Dijon) and the Jean Minjoz University Hospital (Besançon) and included adult patients treated with a curative intent for stage I-III (T1-4 N0-3 M0) resectable HPV-OPSCC between 2010 and 2021.

Patients were excluded from this analysis if they presented with any of the following criteria:

- a past medical history of cancer of any kind and/or of radiation therapy in the head and neck region;
- 75 years old at the onset of treatment;
- unresectable or stage IV disease or neck metastases of unknown primary origin;
- not fit for surgery;
- non-HPV-OPSCC.

The stage of the disease was determined in accordance with TNM American Joint Committee on Cancer (AJCC) 8^th^ edition for HPV-related OPSCCs [16]. For patients whose initial staging was performed using the 7^th^ edition, a new staging according to the 8^th^ edition was retrospectively performed.

Comorbidities were evaluated through the modified Charlson Comorbidities Index (mCCI).

### Determination of HPV status

HPV evaluation could be carried out using immunohistochemistry (IHC) staining for p16 or by performing HPV-specific testing (DNA, RNA or in situ hybridization) [7]. If at least one of the aforementioned tests was positive, the carcinoma was considered HPV-related.

### Resectability

The resectability of the tumor was evaluated by the multidisciplinary tumor board (MTB). If no clear mention of it was found in the medical file of a patient who met the other inclusion criteria, resectability through an open surgical procedure was retrospectively evaluated by a trained head and neck surgical oncologist.

### Data extraction

Data were extracted from the medical information department of each facility using the International Classification of Diseases (ICD). A comprehensive search of each patient’s medical records was conducted from 01/02/2022 to 03/31/2022. Although the authors had access to patients’ identification during the study of the medical records, extracted data was subsequently anonymized prior to analysis.

### Treatments

Patients were treated in accordance with international guidelines [7, 8]. The therapeutic strategy was discussed by the MTB and with the patients.

For the purposes of this study, patients were divided into two groups: those who underwent oncologic surgery followed by adjuvant RT or RCT if needed (uS group) and those who received exclusive RT or RCT (eRT±CT group).

In patients who underwent surgery, postoperative radiotherapy was performed if at least one of the following risk factors was identified: (1) pT3-4, (2) positive surgical margins, (3) perineural infiltration and lymphovascular spread, (3) invaded lymph nodes. Chemotherapy was associated to adjuvant RT in case of (1) positive surgical margins and/or (2) extracapsular node infiltration.

### Data collection

#### Efficacy criteria

Treatment efficacy was evaluated through the following criteria: progression-free survival (PFS), overall survival (OS), disease-specific survival (DSS), locoregional progression-free survival (LRPFS) and metastasis-free survival (MFS). PFS was defined as the time from treatment to disease recurrence, progression or death of any cause. OS represented the time from treatment to death of any cause. DSS accounted for the time from treatment to death from OPSCC. LRPFS and MPFS were defined as the time from treatment to local and/or regional recurrence and metastatic relapse, respectively.

We also reported the patterns of relapse.

#### Tolerance and toxicity assessment

Toxicity was evaluated by physicians during follow-up according to the Common Terminology Criteria for Adverse Events (CTCAE) version 5.0. Acute and late toxicities were defined as toxicities occurring within 90 days after treatment or beyond, respectively.

Data concerning the hospital admissions of patients during treatment, the use of opioids in the management of pain and the need for a feeding tube were also collected.

#### Quality of life assessment

QoL was transversally evaluated using the European Organization for Research and Treatment of Cancer (EORTC) QLQ-C30 [17] and QLQ-H&N35 [18] instruments.

The EORTC QLQ-C30 contains five functional scales (physical, role, cognitive, emotional, and social functioning), a global QoL scale, three symptom scales (fatigue, nausea and vomiting, and pain), and six single items (appetite loss, diarrhea, dyspnea, constipation, insomnia and financial impact). The questionnaire uses a four-point response format (“not at all,” “a little,” “quite a bit,” and “very much”), with the exception of the global QoL scale, which has a seven-point response format. The EORTC QLQ-H&N35 is divided into six scales (pain, swallowing, nutrition, speech, social function, body image, and sexuality) and uses the same four-point response scale as the QLQ-C30.

A high score for a functional scale represents a high level of functioning, a high score for the global health status represents a high QoL, but a high score for a symptom item represents a high level of symptomatology.

These questionnaires were sent by mail in March 2022 to each living patient who was included in the retrospective analysis. Those who did not reply received the questionnaires again in May 2022.

### Statistical analysis

Descriptive analyses and QoL scores were performed using means with standard deviations for quantitative variables and percentages for qualitative variables. Comparison between the groups (uS *versus* eRT±CT) was performed using Student’s t test, Wilcoxon‒Mann‒Whitney, CHI^2^ or Fisher’s test as appropriate.

Survival rates and medians were determined using the Kaplan‒Meier method. Survival curves were compared with the log-rank test. Median follow-up was determined using the reverse Kaplan‒Meier method.

All tests were two-sided. P values less than 5% were considered significant. Analyses were performed using SAS 9.4 software.

This study was approved by the Patient Protection Commission of Ile de France VII on January 12, 2022 (file number: 21.03627.000061).

## Results

### Patients

Between 2010 and 2021, we identified 83 patients who met the inclusion criteria and therefore were included in this study: 64 (77.1%) male and 19 (22.9%) female patients. The main characteristics of the patients are reported in **Table 1**.

**Table 1:** Demographic and clinical characteristics of the patients.

Characteristics		All patients	uS group	eRT±CT group	p value
		n = 83	n = 21	n = 62	
**Sex**					**0,23**
	female	19 (22,9%)	7 (33,3%)	12 (19,4%)	
	male	64 (77,1%)	14 (66,6%)	50 (80,6%)	
**Mean age** (SD), years old					**0,82**
		60,6 (9,6)	60,6 (10,9)	60,5 (9,1)	
**Performance Status** (WHO)					**0,02**
	0	9 (11,1%)	1 (4,8%)	8 (12,9%)	
	1	53 (65,4%)	9 (42,9%)	44 (71,0%)	
	2	18 (22,2%)	8 (38,1%)	10 (16,1%)	
	3	1 (1,2%)	1 (4,8%)	0	
**Mean modified Charlson comorbidities index** (SD)					**0,60**
		4,2 (2,1)	4,5 (2,60)	4 (1,51)	
**Tobacco consumption**, pack-years					**0,45**
	< 10	40 (48,2%)	12 (57,1%)	28 (45,2%)	
	> 10	43 (51,8%)	9 (42,9%)	34 (54,8%)	
**Alcohol consumption**					**1**
	Yes	19 (22,9%)	5 (23,8%)	14 (22,6%)	
	No	64 (77,1%)	16 (76,2%)	48 (77,4%)	
**Hypertension**					**1**
	Yes	22 (26,5%)	5 (23,8%)	17 (27,4%)	
	No	61 (73,5%)	16 (76,2%)	45 (72,6%)	
**Diabetes**					**0,26**
	Yes	4 (4,8%)	2 (9,5%)	2 (3,2%)	
	No	79 (95,2%)	19 (90,5%)	60 (96,8%)	
**Ischemic heart disease**					**1**
	Yes	11 (13,3%)	3 (14,3%)	8 (12,9%)	
	No	72 (86,7%)	18 (85,7%)	54 (87,1%)	
**T classification**					**0,10**
	missing	1 (1,2%)	1 (5%)	0	
	1	10 (12,0%)	4 (20,0%)	6 (9,7%)	
	2	39 (47,0%)	11 (55,0%)	28 (45,2%)	
	3	19 (22,9%)	1 (5,0%)	18 (29,0%)	
	4	14 (16,9%)	4 (20,0%)	10 (16,1%)	
**N classification**					**0,60**
	0	12 (14,4%)	4 (19,0%)	8 (12,9%)	
	1	54 (65,1%)	15 (71,4%)	39 (62,9%)	
	2	11 (13,3%)	1 (4,8%)	10 (16,1%)	
	3	6 (7,2%)	1 (4,8%)	5 (8,1%)	
**Tumor stage**					**0,002**
	I	28 (33,7%)	13 (61,9%)	15 (24,2%)	
	II	31 (37,4%)	2 (9,5%)	29 (46,8%)	
	III	24 (28,9%)	6 (28,6%)	18 (29%)	
**Tumoral site**					**0,45**
	palatine tonsil or glosso-tonsillar sulcus	51 (61,4%)	15 (71,4%)	36 (58,1%)	
	Base of tongue	24 (28,9%)	5 (23,8%)	19 (30,6%)	
	Vallecula	4 (4,8%)	0	4 (6,5%)	
	Posterior pharyngeal wall	2 (2,4%)	0	2 (3,2%)	
	Lateral pharyngeal wall	1(1,2%)	1 (4,8%)	0	
	Soft palate	1 (1,2%)	0	1 (1,6%)	
**Treatments**					
**Radiotherapy**					**N/A**
	Yes	83 (100%)	21 (100%)	62 (100%)	
	No	0	0	0	
**Chemotherapy**					**0,17**
	Yes	69 (83,1%)	15 (71,4%)	54 (87,1%)	
	No	14 (16,9%)	6 (28,6%)	8 (12,9%)	

uS: upfront surgery, eRT±CT: exclusive radiotherapy ± chemotherapy, SD: standard deviation, WHO: World Health Organization, N/A: not applicable

The mean age at diagnosis was 60.6 ± 9.6 years. The most frequent localizations were the palatine tonsil or glosso-tonsillar sulcus (61.4%) and the base of the tongue (28.9%).

Notably, patients significantly differed between the treatment groups in terms of performance status (p = 0.02) and overall tumor stage (p = 0.002).

The evaluation of HPV status was carried out using IHC staining for p16 in 70 (84.3%) patients, HPV DNA testing in five (6.1%) patients, and both methods in 8 (9.6%) patients.

### Treatments

Twenty-one (25.3%) patients received uS, whereas 62 (74.7%) were treated with eRT±CT.

All patients in the uS group underwent a non-minimally invasive surgery, either open or transorals. Surgical details are described in **S1 Table**. Of the 21 patients of this group, 10 (47.6%) had a histologically complete resection (R0), 11 (53.2%) had a R1 resection. Lymphatic emboli and perineural neoplastic invasion were identified in 6 (29%) and 7 (33%) patients, respectively. Sixteen (76%) patients underwent a lymph node dissection, either unilateral (11 (68.7%)) or bilateral (5 (31.3%)). Among them, four were pN0, 10 were pN1, two were pN2 and five had at least one adenopathy with capsular rupture features. All patients received adjuvant treatments following surgery: 15 (71.4%) had RCT, and 6 (28.6%) received RT alone. In the nonsurgical group, 54 (87.1%) patients received CT in addition to RT, while eight (12.9%) received eRT.

In the uS group, the median prescribed dose to the tumor bed and the involved lymph nodes (CTV1) and to the uninvolved lymphatics (CTV2) was 66 (60-66) Gy and 52.8 (50-52.8) Gy, respectively. In the eRT±CT group, the median prescribed dose to the tumor and the involved lymph nodes (CTV1) and to the uninvolved lymphatics (CTV2) was 70 (70-70) Gy and 56 (50-56) Gy, respectively. All patients received a prophylactic irradiation of the uninvolved lymphatics. Bilateral irradiation was delivered to 21 (71.4%) patients and 55 (88.7%) patients in the uS and eRT±CT groups, respectively. In both groups, all patients received intensity-modulated radiation therapy, either volumetric-modulated arc therapy (89.2%) or static step-and-shoot IMRT (10.8%).

When administered, CT was mostly concurrent with RT (95.5%), with cisplatin being the most commonly used drug (85.6%), followed by carboplatin plus 5-fluorouracil (8.7%). Chemotherapy regimens and doses are detailed in **S2 Table**.

### Efficacy outcomes

**Fig 1:**
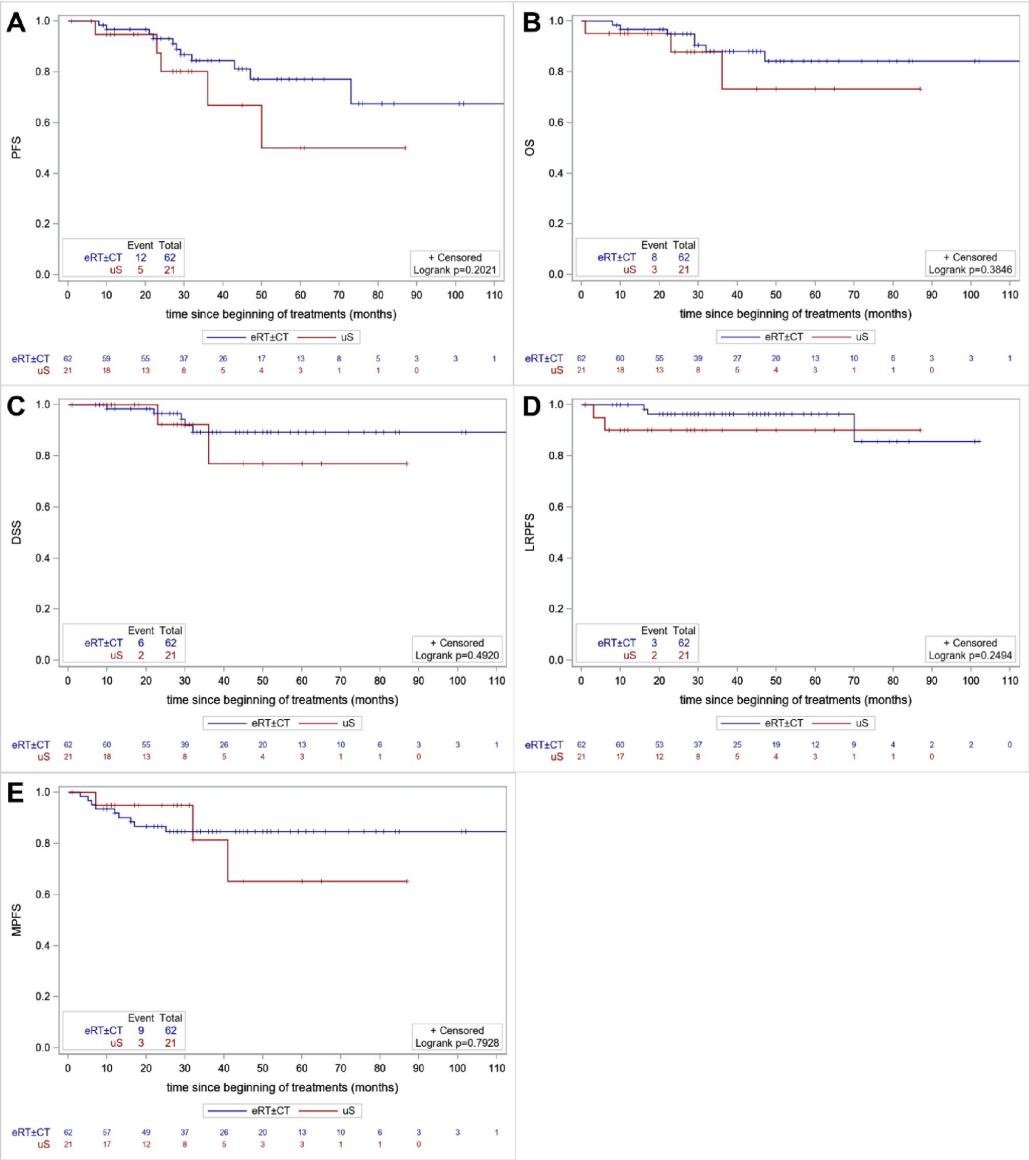
Kaplan‒Meier estimates of PFS (A), OS (B), DSS (C), LRPFS (D) and PFS (E). All stages (I-III). PFS: progression-free survival, OS: overall survival, DSS: disease-specific survival, LRRFS: locoregional progression-free survival, MFS: metastasis-free survival.

**All stages.** The median follow-up in the cohort was 37.0 months. PFS, OS, DSS, LRPFS and MPFS are presented as Kaplan‒Meier curves in **Fig 1** and as survival rates in **Table 2**.

**Table 2:** Oncological outcomes at 2 and 3 years.

Tumor stage	Therapeutic strategy	2-year rates				
		PFS	OS	DSS	LRPFS	MPFS
**All stages (I-III)**						
** **	uS	80%	86%	91%	90%	95%
** **	eRT±CT	92%	95%	96%	95%	85%
**Early stages (I-II)**						
** **	uS	87%	100%	100%	87%	100%
** **	eRT±CT	95%	98%	100%	97%	93%
**Locally advanced stage (III)**						
** **	uS	67%	56%	67%	100%	78%
** **	eRT±CT	84%	87%	87%	100%	68%
		**3-year rates**				
** **		PFS	OS	DSS	LRPFS	MPFS
**All stages (I-III)**						
** **	uS	67%	73%	78%	90%	80%
** **	eRT±CT	84%	86%	89%	95%	85%
**Early stages (I-II)**						
** **	uS	87%	100%	100%	87%	100%
** **	eRT±CT	90%	92%	97%	97%	93%
**Locally advanced stage (III)**						
** **	uS	37%	56%	56%	100%	40%
** **	eRT±CT	58%	75%	62,2%	100%	58%

PFS: progression-free survival, OS: overall survival, DSS: disease-specific survival, LRPFS: locoregional progression-free survival, MPFS: metastatic progression-free survival, uS: upfront surgery, eRT±CT: exclusive radiotherapy ± chemotherapy, N/A: not applicable

There was no significant difference in PFS between the treatment groups (p = 0.20). PFS at two and three years was 80% and 67% in the uS group and 92% and 84% in the eRT±CT group. Predictive factors for PFS are available in **S3 Table**. Alcohol consumption (HR = 3.07, p = 0.03, 95% CI [1.139;8.272]) and stage III disease (*versus* stage I-II, HR = 5.563, p = 0.001, 95% CI [1.996;15.506]) were significantly associated with a worsening PFS contrary to tobacco consumption, Charlson index, age and treatment strategy.

Similarly, there was no difference in OS between the surgical and nonsurgical groups (p = 0.38). The 2- and 3-year OS rates were 86% and 73% in the uS group and 95% and 86% in the eRT±CT group, respectively.

We did not find any significant difference in terms of DSS, LRPFS or MPFS depending on the treatment groups.

### Early stages (I-II)

No significant difference was found in PFS (p = 0.55), OS (p = 0.39), DSS (p = 0.65), LRPFS (p = 0.20) or MPFS (p = 0.32). Data are presented in **Table 2**.

### Locally advanced stage (III)

No significant difference was found in PFS (p = 0.61), OS (p = 0.23), DSS (p = 0.54), LRPFS (p = 1) or MPFS (p = 0.39). The data are shown in **Table 2**.

### Patterns of relapse

**Fig 2:**
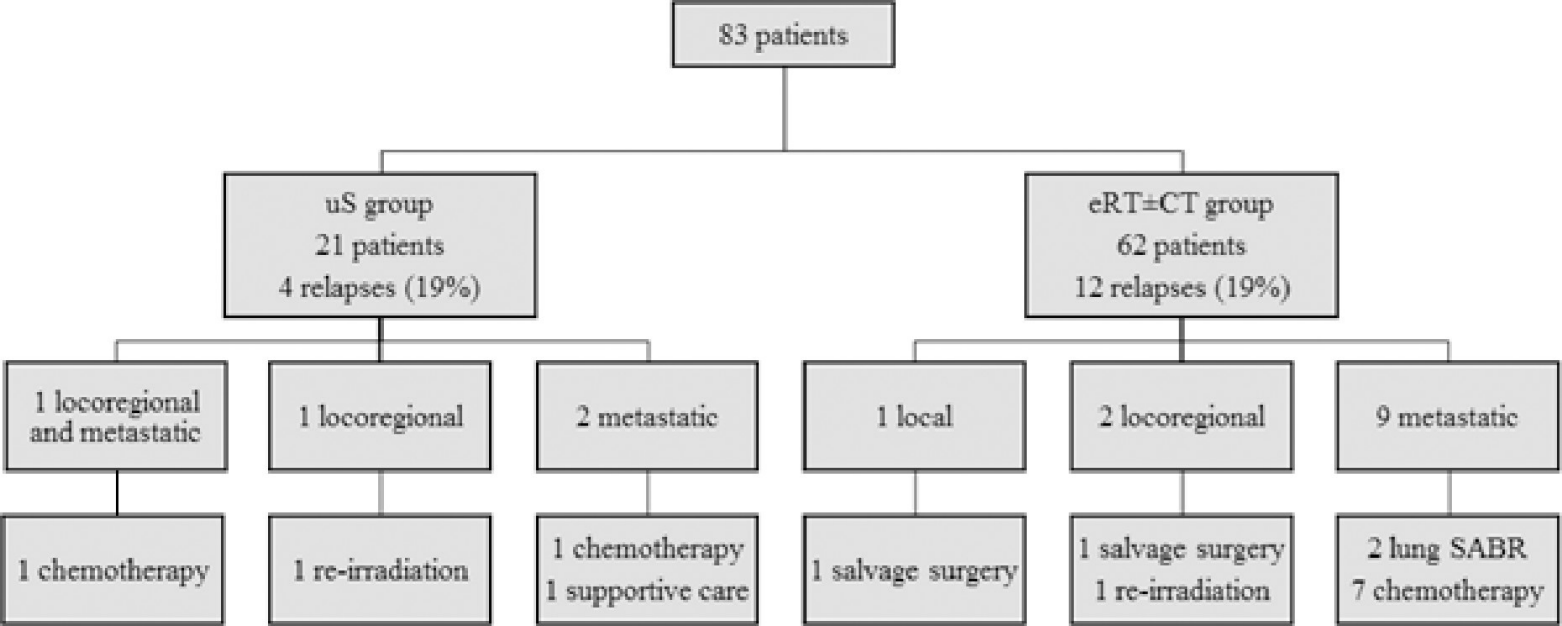
Flow diagram outlining patterns of relapse in both groups. uS: upfront surgery, eRT±CT: exclusive radiotherapy ± chemotherapy, SABR: stereotactic ablative radiotherapy.

During the follow-up period, 16 (19%) patients presented a relapse. Patterns of relapse are presented in **Fig 2**.

In patients with local or locoregional recurrence in the eRT±CT group, two underwent salvage surgery, and one was reirradiated. Two patients developed locoregional failure after uS. The first patient underwent reirradiation, while the second patient underwent chemotherapy because of concurrent metastatic relapse.

In patients with a metastatic relapse, two presented an oligometastatic pulmonary progression treated by stereotactic ablative radiotherapy (SABR), nine had chemotherapy and one received supportive care.

### Tolerance and clinician-reported toxicities

#### Treatment tolerance and discontinuation

There was no treatment-related death in either group. One patient presented postoperative bleeding which required a surgical revision.

In both groups, every patient received the full planned RT dose.

Among those with an indication for CT, 37 (54%) received the treatment as planned, and 32 (46%) prematurely discontinued it. Causes for discontinuation were chemotherapy-related toxicity (88.4%), sepsis (7.8%) and patient refusal to continue CT (3.8%). There was no significant difference in CT discontinuation between the groups (uS group: 8 (53%); eRT±CT group: 29 (54%), p = 1).

*Acute toxicities*. At the end of treatments, 52 (63.4%) patients presented at least one grade III side effect: 15 (75%) after uS and 37 (59.7%) in the eRT±CT group. The most common grade III symptoms were dysphagia (47 (57.3%)) and oral mucositis (12 (14.6%)). Notably, patients presented significantly more oral mucositis in the surgical group, with 17 (85.0%) presenting grade II-III oral mucositis *versus* 33 (53.2%) in the nonsurgical group (p= 0.009). There was no significant difference between the treatment groups concerning other symptoms and all-type toxicity. Data are reported in Table 3.

**Table 3:** Physician-reported toxicities at the end of treatments.

Toxicity		uS		eRT±CT			p value	
	Grade	n = 20	%		n = 62	%		
**All-type maximum toxicity**								**0.15**
** **	0	1	5,00%		0	0,0%		
** **	1	0	0,0%		1	1,6%		
** **	2	4	20,00%		24	38,7%		
** **	3	15	75,00%		37	59,7%		** **
**Dysphagia**								**0.48**
** **	0	0	0,0%		1	1,6%		** **
** **	1	1	5,00%		10	16,1%		** **
** **	2	4	20,00%		18	29,0%		** **
** **	3	14	70,00%		33	53,2%		** **
** **	missing	1	5,00%		0	0,0%		** **
**Odynophagia**								**0.22**
** **	0	0	0,0%		1	1,6%		** **
** **	1	4	20,00%		25	40,3%		** **
** **	2	12	60,00%		31	50,0%		** **
** **	3	2	10,00%		2	3,2%		** **
** **	missing	2	10,00%		3	4,8%		** **
**Xerostomia**								**0.08**
** **	0	1	5,00%		1	1,6%		
** **	1	15	75,00%		18	29,0%		
	2	3	15,00%		36	58,1%		
	3	0	0,0%		5	8,1%		
	missing	1	5,00%		2	3,2%		
**Oral mucositis**								**0.009**
** **	0	0	0%		2	3,2%		** **
** **	1	1	5,0%		24	38,7%		** **
** **	2	14	70,0%		24	38,7%		** **
** **	3	3	15,0%		9	14,5%		** **
** **	missing	2	10,0%		3	4,8%		** **
**Dysgueusia**								**0.40**
** **	0	1	5,0%		7	11,3%		** **
** **	1	6	30,0%		20	32,3%		** **
** **	2	9	45,0%		16	25,8%		** **
** **	missing	4	20,0%		19	30,6%		** **
**Trismus**								**1**
** **	0	17	85,0%		56	90,3%		** **
** **	1	0	0%		3	4,8%		** **
** **	missing	3	15,0%		3	4,8%		** **
**Pain**								**0.85**
** **	0	1	5,0%		3	4,8%		** **
** **	1	8	40,0%		22	35,5%		** **
** **	2	8	40,0%		32	51,6%		** **
** **	3	2	10,0%		5	8,1%		** **
** **	missing	1	5,0%		0	0%		** **
**Radiodermatitis**								**0.60**
** **	0	0	0%		0	0%		** **
	1	4	20,0%		21	33,9%		** **
	2	13	65,0%		36	58,1%		** **
	3	2	10,0%		5	8,0%		** **
	missing	1	5,0%		0	0%		

uS: upfront surgery, eRT±CT: exclusive radiotherapy ± chemotherapy

At six months, one patient still reported grade III pain after uS and adjuvant RCT. No patient in the nonsurgical group had any grade III toxicity. The main grade II symptom was xerostomia (7). No significant difference was found between the surgical and nonsurgical groups in terms of overall toxicity at six months (**S4 Table**), but dysphagia was more frequently reported in the surgical group, although it did not meet statistical significance (grade I-II, 25% *vs* 14.5%, p = 0.06).

### Late toxicities

No grade III toxicity was reported by physicians at 1 and 2 years after treatment. At two years, 2 (22.2%) patients had grade II toxicity in the uS group, and four (8.7%) in the eRT±CT group. Grade II side effects were xerostomia (4), dysphagia (1), and pain (1). No significant difference was found in late toxicity between each group. Data at two years are shown in **S5 Table**.

### Nutritional support

At the end of treatment, a greater proportion of patients had a feeding tube in place in the uS group, although this difference was not significant (14 (66.7%) *vs* 35 (56.4%), p = 0.28). At one year, one patient in the surgical group and four patients in the eRT±CT group were still dependent on a feeding tube.

### Patient-reported quality of life

A total of 45 patients completed both QoL questionnaires: 11 from the uS group and 34 from the nonsurgical group. One patient who received eRCT returned a completed EORTC QLQ-C30 but not the QLQ-H&N35. Data are presented in **S6 and S7 Tables**.

The median time between treatment completion and the response to the questionnaires was 38.4 (Q1 25.7; Q3 56.2) months in the overall population, 17.0 (Q1 8.75; Q3 26.5) in the uS group and 44.7 (Q1 29.6; Q3 60.1) for the eRT±CT group (p < 0.01).

Fatigue (34.3 *vs* 21.0, p = 0.03), appetite loss (33.3 *vs* 8.6, p = 0.005) and the need for nutritional supplements (36% *vs* 6%, p = 0.02) were found to be significantly greater in patients who underwent surgery. However, patients in the eRT±CT group reported more swallowing issues (80.1 *vs* 59.8, p = 0.008), sticky saliva (54.9 *vs* 30.3, p = 0.05), and mouth and teeth problems (80.4 *vs* 57.6, p = 0.04 and 79.4 *vs* 48.5, p = 0.02, respectively).

Role functioning was better in the eRT±CT group, close to statistical significance (91.4 *vs* 80.3, p = 0.06). Physical, cognitive, emotional and social functioning, pain, speech, social function, body image, and sexuality were not significantly different in either group. Finally, the global health status from the QLQ-C30 instrument was better in the nonsurgical group, although this did not reach statistical significance (77.6 *vs* 65.2, p = 0.07).

## Discussion

The purpose of this study was to assess the efficacy, tolerance and impact on quality of life of two therapeutic strategies in the treatment of localized and locally advanced HPV-OPSCCs: upfront surgery followed or not by adjuvant RT±CT and exclusive RT±CT.

Both study groups were quite homogeneous but differed from each other, as patients from the uS group had a higher performance status and those from the eRT±CT group presented a higher tumor stage. However, the proportion of stage III OPSCC was the same in both groups. Tumors that developed within the palatin tonsils or the glosso-tonsillar sulcus were more likely to be treated surgically, even though this was not significant. These elements should be taken into account, as they are likely to affect the efficacy outcomes of both treatment groups even if they did not seem to impact PFS in the predictive factor analysis.

Both strategies were found to be effective in the treatment of stage I-III HPV-OPSCC, with no significant difference in PFS, OS, DSS, LRPFS and MPFS between uS and eRT±CT in the whole population, neither in early stages nor in more advanced diseases. Our results seem consistent with the sparse data available in the literature. In a retrospective study of over 3,000 patients with stage I-II HPV-OPSCC [13], Kelly *et al*. found no difference in OS between a surgical approach and eRCT as did Kim *et al*. in a more recent series [19]. In locally advanced tumors - stage III-IV according to the AJCC 7^th^ version - Kamran *et al*. reported an OS benefit from surgery over RT-CT [20]. This retrospective study did not focus only on HPV-OPSCC, although clinical characteristics were not available for the 5,037 patients with an HPV-related tumor. Additionally, OS was not significantly different between the treatment groups in the multivariate analysis. This suggests that confounding factors may have influenced the difference in OS observed in the univariate analysis. These results came from American databases which enable the collection of data on large populations. However, the only provide information on overall survival, and data about other relevant oncological outcomes are missing.

Recently, the GETTEC (Groupe d’Étude des Tumeurs de la Tête Et du Cou) group conducted a study on 382 patients with HPV-OPSCC in seven French centers [15]. There was no difference in OS at five years (89.2% and 84.2% in the surgical and nonsurgical groups, respectively), but DSS and RFS were found to be improved in the surgical group in the multivariate analysis. Our study yielded comparable similar survival rates and patterns of relapse to those published by Culié *et al*. (19% of relapse in our study *vs* 20%) [15], yet we did not find any significant difference in efficacy between the two groups. Despite the limited number of events and the presence of confounding factors, it may traduce good outcomes in our eRT±CT group. Lacau St Guily *et al*. also found no difference in RFS at two years after uS or eRT±CT in 92 patients with HPV-OPSCC [21].

A meta-analysis was conducted to evaluate both strategies, irrespective of HPV status [22]. The authors found a significant heterogeneity between studies and concluded that OS after surgical and nonsurgical treatments was not significantly different. However, this meta-analysis mostly comprised unicentric single-modality studies with a small number of patients, highlighting the importance of data from well-conducted large retrospective and prospective studies.

Because patients with HPV-OPSCCs have better outcomes compared to those with non-HPV-related tumors [5, 23], the issue of treatment tolerance and QoL has become increasingly important [24]. Notably, all patients from our study received IMRT, as it has been demonstrated to reduce the incidence of xerostomia and improve saliva-related QoL compared to 3D RT [25, 26]. Overall acute and late toxicities did not significantly differ between the treatment groups and the incidence of severe adverse events was low, with no grade IV-V side effects reported.

In our series, patients in the surgical group received either non-robotic transoral surgery or open surgery. One potential avenue for reducing treatment-related toxicity is minimally invasive procedures, such as transoral robotic surgery (TORS). However, the ORATOR phase II study, which compared QoL after TORS with or without adjuvant treatments to eRT±CT in the treatment of T1-2 N0-2 OPSCCs [10], found a trend for more grade III dysphagia in the surgery group (26% *vs* 18%), as in our study, even though the surgical technique was minimally invasive and only 74% of patients received adjuvant RT in the surgical group [10]. Moreover, the ORATOR2 trial comparing TORS ± 50-60 Gy RT and 60 Gy RT plus weekly cisplatin in Ti-2 N0-2 tumors focusing on efficacy was prematurely discontinued due to excessive toxic effects in the surgical arm [27]. Long-term outcomes are awaited with this surgical approach, but caution is required outside expert centers.

It is well established that QoL and toxicity assessment by physicians leads to a lower incidence and severity of symptoms compared to patient reports [28]. Therefore, we decided to collect patient-reported outcomes in addition to data retrospectively extracted from medical files. We used the multi-item multidomain EORTC QLQ-C30 and QLQ-H&N35 instruments, which are valid measurement tools for QoL having been used in many international clinical trials [29–32]. Additionally, they have been shown to be predictive of OS in multiple studies [33–37]. As the questionnaires were sent to alive patients at a specific time point, the median time between treatment and response to the questionnaires was significantly different between the two groups. It is important to note that the results obtained are purely descriptive and do not allow us to conclude that one treatment arm is superior to the other in terms of safety, as timing is an obvious confounding factor. We did note that the toxicity profiles appeared to differ between groups: patients in the eRT±CT group reported more swallowing issues and sticky saliva, whereas those in the uS group seemed to experience more fatigue and appetite loss.

OPSCC survivors face clinically important deteriorations in QoL mostly centered on xerostomia, dysphagia and chewing [24, 38]. Patients with an HPV-positive tumor have their own course of QoL during and after treatment [39], but studies comparing surgical and nonsurgical strategies are scarce in this particular population. Yin *et al*. showed a worse QoL after surgery alone or upfront surgery followed by RT compared to eRT at three and six months after treatment [40]. Unfortunately, no long-term data were available. In addition, longitudinal swallowing QoL according to the MD Anderson Dysphagia Inventory (MADI) score was significantly superior with RT in the ORATOR trial [10, 41]. Nevertheless, this discrepancy did not meet the predefined threshold of a clinically relevant change in QoL and was no longer significant at two and three years.

In our study, no patient underwent surgery without any adjuvant treatment, possibly due to a selection bias. Thus, our findings cannot be extrapolated to pT1-2 pN0-N1 tumors with negative margins and no criteria for adjuvant treatments. Nevertheless, 97% of the patients in the GETTEC study [15] and two-thirds of the patients in the ORATOR trial [10] received adjuvant therapies, suggesting that only a small number of patients are treated by surgery alone. This implies that patients undergoing surgery are likely to require a multimodal approach which may increase the risk of toxicity and deteriorate QoL. Patients must be carefully informed of the oncological outcomes of uS and eRT±CT and of the high rate of adjuvant treatments after surgery. Again, selecting the population that might benefit from surgery alone as well as finding effective treatment de-escalation are consequently paramount.

Our work has several limitations. First, oncological data and physician-reported toxicities were retrospectively obtained, which introduces inherent flaws into this retrospective series. Moreover, because of a lack of power due to very few events, only a univariate analysis could be performed. Therefore, our results might have been influenced by some confounding factors. However, our study population was quite homogeneous between the treatment groups and did not differ in terms of alcohol consumption and stage III disease, the only two variables that were found to impact PFS. This suggests that the effects of potential confounding factors are limited. Finally, five patients in the surgical group did not undergo lymph node dissection. As retrospective series aims to present data representative of real-world clinical practice, we decided to include these patients in the analysis. Kim et al. [19] also reported that six patients in their series had not undergone a lymph node dissection, while the series by Kelly et al. [13] and Kamran et al. [20] did not specify whether a lymph node dissection had been performed. It is possible that suboptimal surgery may have contributed to a lower efficacy in the surgical arm. However, in our series, only one of the five patients with no lymph node dissection presented with a recurrence.

This work also has strengths. It is indeed, to the best of our knowledge, one of the largest European studies in this field of research and one of the rare to focus not only on efficacy but also on tolerance. The multicentric nature of this study serves to reduce the impact of center effects on the outcomes observed. Additionally, data were collected at three academic centers with expertise in the management of OPSCC and that follow international guidelines, leading to meaningful results.

Therefore, our findings provide novel insights into the management of HPV-OPSCC in the era of therapeutic de-escalation and may help physicians find the most suitable treatment strategy for each individual. It is unlikely that phase III trials comparing surgery and radiotherapy will ever be conducted due to difficulties in recruitment. However, the debate between surgery and radiotherapy could be clarified by large, international prospective studies aiming to identify predictive factors for one strategy or the other.

## Conclusion

We found comparable oncologic outcomes between upfront surgery and exclusive radiotherapy or radiochemotherapy in the treatment of stage I-III HPV-OPSCC. Notably, all patients who underwent surgery received adjuvant treatments. Acute and late toxicity were not significantly different between the treatment groups. The transversal exploratory quality of life analysis showed that tolerance profiles could differ between the treatment groups.

These results must be validated through further prospective studies. In the interim, patients should be informed of these elements by physicians before making a decision regarding the treatment strategy.

## Supporting information

S1 Table Surgical characteristics of patients in the uS group. uS: upfront surgery.
(DOCX)

S2 Table Characteristics of the chemotherapy regimen. Us: upfront surgery, eRT±CT: exclusive radiotherapy ± chemotherapy, 5FU: 5 fluorouracil.
(DOCX)

S3 Table Predictive factors for PFS. PFS: progression-free survival, eRT±CT: exclusive radiotherapy ± chemotherapy, uS: upfront surgery.
(DOCX)

S4 Table Physician-reported toxicities at 6 months. uS: upfront surgery, eRT±CT: exclusive radiotherapy ± chemotherapy.
(DOC)

S5 Table Physician-reported toxicities at 24 months. uS: surgery, eRT±CT: exclusive radiotherapy ± chemotherapy, N/A: not applicable.
(DOC)

S6 Table Patient-reported quality of life – EORTC QLQ-C30 instrument. uS: upfront surgery, eRT±CT: exclusive radiotherapy ± chemotherapy, SD: standard deviation.
(DOC)

S7 Table Patient-reported quality of life – EORTC QLQ-H&N35 instrument. uS: upfront surgery, eRT±CT: exclusive radiotherapy ± chemotherapy, SD: standard deviation.
(DOC)
