# Supplementary material for: Definitive radio(chemo)therapy versus upfront surgery in the treatment of HPV-related localized or locally advanced oropharyngeal squamous cell carcinoma
Source: PLoS One. 2024 Jul 25;19(7):e0307658. doi: 10.1371/journal.pone.0307658 (PMC11271858; doi:10.1371/journal.pone.0307658)
Supplement: S1 Table — uS: upfront surgery. (DOCX) [file pone.0307658.s001.docx]

**S1 Table. Surgical characteristics of patients in the uS group**

*uS: upfront surgery*

| Patients | Surgical procedure | Lymph node dissection |
| --- | --- | --- |
| 1 | Transoral lateral oropharyngectomy | - |
| 2 | Transoral lateral oropharyngectomy | - |
| 3 | Transmandibular buccopharyngectomy | Unilateral |
| 4 | Transoral lateral oropharyngectomy | Unilateral |
| 5 | Transoral lateral oropharyngectomy | Unilateral |
| 6 | Transmandibular buccopharyngectomy | Unilateral |
| 7 | Transoral lateral oropharyngectomy | Unilateral |
| 8 | Transmandibular buccopharyngectomy | Unilateral |
| 9 | Transmandibular buccopharyngectomy | Unilateral |
| 10 | Transoral lateral oropharyngectomy | Unilateral |
| 11 | Transoral lateral oropharyngectomy | - |
| 12 | Transoral lateral oropharyngectomy | Unilateral |
| 13 | Transoral lateral oropharyngectomy | - |
| 14 | Transoral lateral oropharyngectomy | - |
| 15 | Transmandibular buccopharyngectomy | Unilateral |
| 16 | Transmandibular buccopharyngectomy | Bilateral |
| 17 | Transmandibular buccopharyngectomy | Bilateral |
| 18 | Transoral lateral oropharyngectomy | Unilateral |
| 19 | Transmandibular buccopharyngectomy | Bilateral |
| 20 | Transmandibular buccopharyngectomy | Bilateral |
| 21 | Transoral lateral oropharyngectomy | Bilateral |
