# Supplementary material for: Definitive radio(chemo)therapy versus upfront surgery in the treatment of HPV-related localized or locally advanced oropharyngeal squamous cell carcinoma
Source: PLoS One. 2024 Jul 25;19(7):e0307658. doi: 10.1371/journal.pone.0307658 (PMC11271858; doi:10.1371/journal.pone.0307658)
Supplement: S2 Table — Us: upfront surgery, eRT±CT: exclusive radiotherapy ± chemotherapy, 5FU: 5 fluorouracil. (DOCX) [file pone.0307658.s002.docx]

**S2 Table. Characteristics of the chemotherapy regimen**

*Us: upfront surgery, eRT±CT: exclusive radiotherapy ± chemotherapy, 5FU: 5 fluorouracil*

| Chemotherapy regimen | Dose | All patients  n=69 | uS group  n=15 | eRT±CT group  n=54 |
| --- | --- | --- | --- | --- |
| Concurrent 3-weekly cisplatin | 100 mg.m^-^² | 59 | 14 | 45 |
| Concurrent weekly cisplatin | 40 mg.m^-^² | 1 | 1 | 0 |
| Concurrent 3-weekly carboplatin + 5FU | Carboplatin: 70 mg.m^-^² for 4 days  5FU: 600 mg.m^-^² for 4 days | 6 | 0 | 6 |
| Concurrent weekly cetuximab | 400 mg. ^-^² at first injection then 250 mg.m^-^² | 1 | 0 | 1 |
| Induction hydroxycarbamide + 5FU | Hydroxycarbamide: 1500 mg  5FU: 800 mg/m² | 1 | 0 | 1 |
| Induction docetaxel + cisplatin + 5FU | Docetaxel: 75 mg.m^-^²  Cisplatin: 75 mg.m^-^²  5FU : 750 mg.m ^-^² | 2 | 0 | 2 |
