# Supplementary material for: Definitive radio(chemo)therapy versus upfront surgery in the treatment of HPV-related localized or locally advanced oropharyngeal squamous cell carcinoma
Source: PLoS One. 2024 Jul 25;19(7):e0307658. doi: 10.1371/journal.pone.0307658 (PMC11271858; doi:10.1371/journal.pone.0307658)
Supplement: S3 Table — PFS: progression-free survival, eRT±CT: exclusive radiotherapy ± chemotherapy, uS: upfront surgery. (DOCX) [file pone.0307658.s003.docx]

**S3 Table. Predictive factors for PFS**

*PFS: progression-free survival, eRT±CT: exclusive radiotherapy ± chemotherapy, uS: upfront surgery*


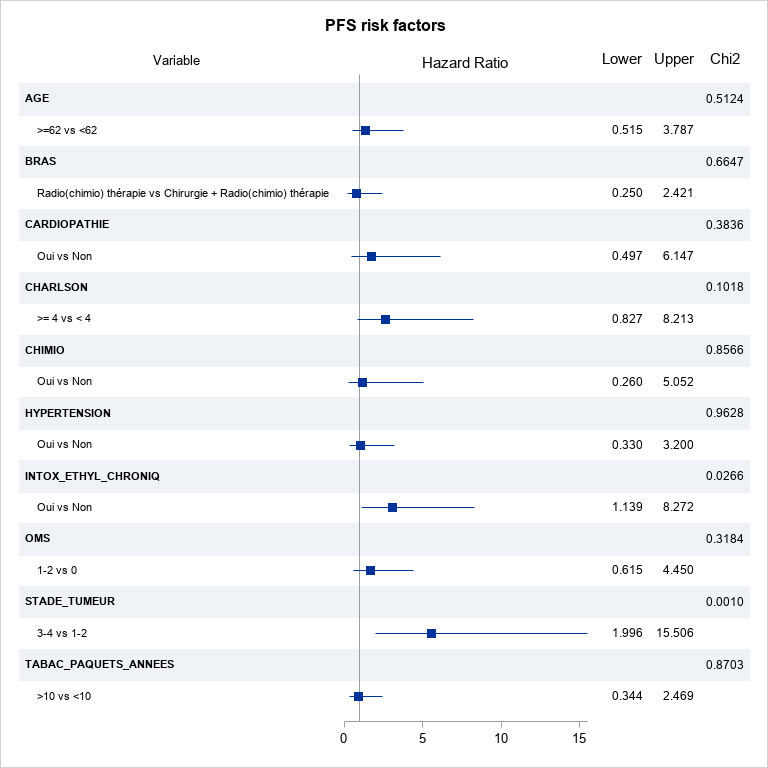


**AGE**

**TREATMENT GROUP**

**ISCHEMIC HEART DISEASE**

**mCHARLSON INDEX**

**CHEMOTHERAPY**

**HYPERTENSION**

**ALCOHOL CONSUMPTION**

**PERFORMANCE STATUS**

**TUMOR STAGE**

**TOBACCO CONSUMPTION**

≥62 vs <62 years

eRT±CT vs uS

Yes vs No

≥4 vs <4

Yes vs No

Yes vs No

Yes vs No

1-2 vs 0

III vs I-II

>10 vs <10 pack-years
