# Supplementary material for: Definitive radio(chemo)therapy versus upfront surgery in the treatment of HPV-related localized or locally advanced oropharyngeal squamous cell carcinoma
Source: PLoS One. 2024 Jul 25;19(7):e0307658. doi: 10.1371/journal.pone.0307658 (PMC11271858; doi:10.1371/journal.pone.0307658)
Supplement: S4 Table — uS: upfront surgery, eRT±CT: exclusive radiotherapy ± chemotherapy. (DOC) [file pone.0307658.s004.doc]

**S4 Table.** **Physician-reported toxicities at 6 months**

*uS: upfront surgery, eRT±CT: exclusive radiotherapy ± chemotherapy*

| **Toxicity** |  | **uS** | | **eRT±CT** | | **p-value** |
| --- | --- | --- | --- | --- | --- | --- |
|  | Grade | n=16 | % | n=55 | % |  |
| **All-type maximum toxicity** |  |  |  |  |  | **0.11** |
|  | 0 | 0 | 0,0% | 5 | 9,1% |  |
|  | 1 | 11 | 68,8% | 43 | 78.2% |  |
|  | 2 | 4 | 25,0% | 7 | 12,7% |  |
|  | 3 | 1 | 6,2% | 0 | 0,0% |  |
| **Dysphagia** |  |  |  |  |  | **0.06** |
|  | 0 | 12 | 75,0% | 47 | 85,5% |  |
|  | 1 | 2 | 12,5% | 8 | 14,5% |  |
|  | 2 | 2 | 12,5% | 0 | 0,0% |  |
|  | missing | 0 | 0,0% | 0 | 0,0% |  |
| **Odynophagia** |  |  |  |  |  | **0.09** |
|  | 0 | 12 | 75,0% | 50 | 90,9% |  |
|  | 1 | 3 | 18,8% | 5 | 9,1% |  |
|  | 2 | 1 | 6,2% | 0 | 0,0% |  |
|  | missing | 0 | 0,0% | 0 | 0,0% |  |
| **Xerostomia** |  |  |  |  |  | **0.45** |
|  | 0 | 0 | 0,0% | 7 | 12,7% |  |
|  | 1 | 14 | 87,5% | 42 | 76,4% |  |
|  | 2 | 2 | 12,5% | 5 | 9,1% |  |
|  | missing | 0 | 0,0% | 1 | 1,8% |  |
| **Oral mucositis** |  |  |  |  |  | **0.52** |
|  | 0 | 13 | 81,3% | 49 | 89,1% |  |
|  | 1 | 1 | 6,2% | 2 | 3,6% |  |
|  | 2 | 0 | 0% | 0 | 0% |  |
|  | missing | 2 | 12,5% | 4 | 7,3% |  |
| **Dysgueusia** |  |  |  |  |  | **0.22** |
|  | 0 | 4 | 25,0% | 22 | 40,0% |  |
|  | 1 | 7 | 43,8% | 18 | 32,7% |  |
|  | 2 | 1 | 6,2% | 1 | 1,8% |  |
|  | missing | 4 | 25,0% | 14 | 25,5% |  |
| **Trismus** |  |  |  |  |  | **0.23** |
|  | 0 | 13 | 81,3% | 48 | 87,3% |  |
|  | 1 | 1 | 6,2% | 0 | 0% |  |
|  | 2 | 0 | 0% | 0 | 0% |  |
|  | missing | 2 | 12,5% | 7 | 12,7% |  |
| **Pain** |  |  |  |  |  | **0.44** |
|  | 0 | 12 | 75,0% | 44 | 80,0% |  |
|  | 1 | 3 | 18,8% | 10 | 18,2% |  |
|  | 2 | 0 | 0% | 1 | 1,8% |  |
|  | 3 | 1 | 6,2% | 0 | 0% |  |
|  | missing | 0 | 0% | 0 | 0% |  |
| **Radiodermatitis** |  |  |  |  |  | **1** |
|  | 0 | 16 | 100,0% | 53 | 96,4% |  |
|  | 1 | 0 | 0% | 1 | 1,8% |  |
|  | 2 | 0 | 0% | 1 | 1,8% |  |
|  | missing | 0 | 0% | 0 | 0% |  |
