# Supplementary material for: Definitive radio(chemo)therapy versus upfront surgery in the treatment of HPV-related localized or locally advanced oropharyngeal squamous cell carcinoma
Source: PLoS One. 2024 Jul 25;19(7):e0307658. doi: 10.1371/journal.pone.0307658 (PMC11271858; doi:10.1371/journal.pone.0307658)
Supplement: S5 Table — uS: surgery, eRT±CT: exclusive radiotherapy ± chemotherapy, N/A: not applicable. (DOC) [file pone.0307658.s005.doc]

**S5 Table. Physician-reported toxicities at 24 months**

*uS: surgery, eRT±CT: exclusive radiotherapy ± chemotherapy, N/A: not applicable*

| **Toxicity** |  | **uS** | | **eRT±CT** | | **p-value** |
| --- | --- | --- | --- | --- | --- | --- |
|  | Grade | n=9 | % | n=46 | % |  |
| **All-type maximum toxicity** |  |  |  |  |  | **0.25** |
|  | 0 | 1 | 11,1% | 8 | 17,4% |  |
|  | 1 | 6 | 66,7% | 34 | 73,9% |  |
|  | 2 | 2 | 22,2% | 4 | 8,7% |  |
| **Dysphagia** |  |  |  |  |  | **0.27** |
|  | 0 | 7 | 77,8% | 43 | 93,5% |  |
|  | 1 | 2 | 22,2% | 2 | 4,3% |  |
|  | 2 | 0 | 0% | 1 | 2,2% |  |
|  | missing | 0 | 0,0% | 0 | 0,0% |  |
| **Odynophagia** |  |  |  |  |  | **0.52** |
|  | 0 | 8 | 88,9% | 43 | 93,5% |  |
|  | 1 | 1 | 11,1% | 3 | 6,5% |  |
|  | 2 | 0 | 0% | 0 | 0% |  |
|  | missing | 0 | 0,0% | 0 | 0,0% |  |
| **Xerostomia** |  |  |  |  |  | **0.20** |
|  | 0 | 2 | 22,2% | 13 | 28,3% |  |
|  | 1 | 5 | 55,6% | 31 | 67,4% |  |
|  | 2 | 2 | 22,2% | 2 | 4,3% |  |
|  | missing | 0 | 0,0% | 0 | 0,0% |  |
| **Oral mucositis** |  |  |  |  |  | **N/A** |
|  | 0 | 7 | 77,8% | 45 | 97,8% |  |
|  | 1 | 0 | 0% | 0 | 0% |  |
|  | 2 | 0 | 0% | 0 | 0% |  |
|  | missing | 2 | 22,2% | 1 | 2,2% |  |
| **Dysgueusia** |  |  |  |  |  | **0,39** |
|  | 0 | 3 | 33,3% | 23 | 50,0% |  |
|  | 1 | 4 | 44,4% | 11 | 23,9% |  |
|  | 2 | 0 | 0% | 0 | 0% |  |
|  | missing | 2 | 22,2% | 12 | 26,1% |  |
| **Trismus** |  |  |  |  |  | **N/A** |
|  | 0 | 7 | 77,8% | 44 | 95,7% |  |
|  | 1 | 0 | 0% | 0 | 0% |  |
|  | 2 | 0 | 0% | 0 | 0% |  |
|  | missing | 2 | 22,2% | 2 | 4,3% |  |
| **Pain** |  |  |  |  | 0,0% | **1** |
|  | 0 | 9 | 100% | 43 | 93,5% |  |
|  | 1 | 0 | 0% | 2 | 4,3% |  |
|  | 2 | 0 | 0% | 1 | 2,2% |  |
|  | missing | 0 | 0,0% | 0 | 0,0% |  |
| **Radiodermatitis** |  |  |  | 0,0% | 1 | **1** |
|  | 0 | 9 | 100% | 45 | 97,8% |  |
|  | 1 | 0 | 0% | 1 | 2,2% |  |
|  | 2 | 0 | 0% | 0 | 0,0% |  |
|  | missing | 0 | 0,0% | 0 | 0,0% |  |
