# Supplementary material for: Definitive radio(chemo)therapy versus upfront surgery in the treatment of HPV-related localized or locally advanced oropharyngeal squamous cell carcinoma
Source: PLoS One. 2024 Jul 25;19(7):e0307658. doi: 10.1371/journal.pone.0307658 (PMC11271858; doi:10.1371/journal.pone.0307658)
Supplement: S6 Table — uS: upfront surgery, eRT±CT: exclusive radiotherapy ± chemotherapy, SD: standard deviation. (DOC) [file pone.0307658.s006.doc]

**S6 Table. Patient-reported quality of life – EORTC QLQ-C30 instrument**

*uS: upfront surgery, eRT±CT: exclusive radiotherapy ± chemotherapy, SD: standard deviation*

| **Variable** | **uS** | **eRT±CT** | **p-value** |
| --- | --- | --- | --- |
| **Physical functioning** |  |  | **0,45** |
| Mean (SD) | 88.5 (14.0) | 90.5 (12.6) |  |
| Median [min - max] | 93.3 [53.3 - 100.0] | 100.0 [60.0 - 100.0] |  |
|  |  |  |  |
| **Role functioning** |  |  | **0,05** |
| Mean (SD) | 80.3 (20.8) | 91.4 (14.2) |  |
| Median [min - max] | 83.3 [33.3 - 100.0] | 100.0 [50.0 - 100.0] |  |
|  |  |  |  |
| **Emotional functioning** |  |  | **0,42** |
| Mean (SD) | 76.5 (20.7) | 81.9 (20.7) |  |
| Median [min - max] | 83.3 [50.0 - 100.0] | 91.7 [33.3 - 100.0] |  |
|  |  |  |  |
| **Cognitive functioning** |  |  | **0,23** |
| Mean (SD) | 90.9 (11.5) | 81.0 (22.6) |  |
| Median [min - max] | 100.0 [66.7 - 100.0] | 83.3 [16.7 - 100.0] |  |
|  |  |  |  |
| **Social functioning** |  |  | **0,61** |
| Mean (SD) | 74.2 (36.8) | 85.7 (21.4) |  |
| Median [min - max] | 100.0 [0.0 - 100.0] | 100.0 [0.0 - 100.0] |  |
|  |  |  |  |
| **Global health status** |  |  | **0,07** |
| Mean (SD) | 65.2 (19.3) | 77.6 (14.8) |  |
| Median [min - max] | 66.7 [25.0 - 83.3] | 83.3 [50.0 - 100.0] |  |
|  |  |  |  |
| **Fatigue** |  |  | **0,03** |
| Mean (SD) | 34.3 (14.4) | 21.0 (21.7) |  |
| Median [min - max] | 33.3 [11.1 - 55.6] | 22.2 [0.0 - 88.9] |  |
|  |  |  |  |
| **Nausea and vomiting** |  |  | **0,15** |
| Mean (SD) | 4.5 (7.8) | 3.3 (12.7) |  |
| Median [min - max] | 0.0 [0.0 - 16.7] | 0.0 [0.0 - 66.7] |  |
|  |  |  |  |
| **Pain** |  |  | **0,28** |
| Mean (SD) | 18.2 (17.4) | 15.2 (25.0) |  |
| Median [min - max] | 16.7 [0.0 - 50.0] | 0.0 [0.0 - 100.0] |  |
| **Dyspnea** |  |  | **0,08** |
| Mean (SD) | 3.0 (10.1) | 16.2 (24.7) |  |
| Median [min - max] | 0.0 [0.0 - 33.3] | 0.0 [0.0 - 100.0] |  |
|  |  |  |  |
| **Insomnia** |  |  | **1** |
| Mean (SD) | 30.3 (23.4) | 34.3 (35.7) |  |
| Median [min - max] | 33.3 [0.0 - 66.7] | 33.3 [0.0 - 100.0] |  |
|  |  |  |  |
| **Appetite loss** |  |  | **0,005** |
| Mean (SD) | 33.3 (33.3) | 8.6 (18.7) |  |
| Median [min - max] | 33.3 [0.0 - 100.0] | 0.0 [0.0 - 66.7] |  |
|  |  |  |  |
| **Constipation** |  |  | **0,74** |
| Mean (SD) | 15.2 (22.9) | 15.2 (28.4) |  |
| Median [min - max] | 0.0 [0.0 - 66.7] | 0.0 [0.0 - 100.0] |  |
|  |  |  |  |
| **Diarrhea** |  |  | **0,32** |
| Mean (SD) | 3.0 (10.1) | 9.5 (20.7) |  |
| Median [min - max] | 0.0 [0.0 - 33.3] | 0.0 [0.0 - 100.0] |  |
|  |  |  |  |
| **Financial impact** |  |  | **0,66** |
| Mean (SD) | 15.2 (27.3) | 12.4 (28.1) |  |
| Median [min - max] | 0.0 [0.0 - 66.7] | 0.0 [0.0 - 100.0] |  |
