# Supplementary material for: Definitive radio(chemo)therapy versus upfront surgery in the treatment of HPV-related localized or locally advanced oropharyngeal squamous cell carcinoma
Source: PLoS One. 2024 Jul 25;19(7):e0307658. doi: 10.1371/journal.pone.0307658 (PMC11271858; doi:10.1371/journal.pone.0307658)
Supplement: S7 Table — uS: upfront surgery, eRT±CT: exclusive radiotherapy ± chemotherapy, SD: standard deviation. (DOC) [file pone.0307658.s007.doc]

**S7 Table. Patient-reported quality of life – EORTC QLQ-H&N35 instrument**

*uS: upfront surgery, eRT±CT: exclusive radiotherapy ± chemotherapy, SD: standard deviation*

| **Variable** | **uS** | **eRT±CT** | **p-value** |
| --- | --- | --- | --- |
| **Swallowing** |  |  | **0,008** |
| Mean (SD) | 59.8 (23.2) | 80.1 (19.6) |  |
| Median [min - max] | 58.3 [25.0 - 100.0] | 83.3 [8.3 - 100.0] |  |
|  |  |  |  |
| **Senses** |  |  | **0,07** |
| Mean (SD) | 72.7 (22.7) | 83.3 (28.1) |  |
| Median [min - max] | 66.7 [33.3 - 100.0] | 100.0 [0.0 - 100.0] |  |
|  |  |  |  |
| **Speech** |  |  | **0,16** |
| Mean (SD) | 74.7 (27.3) | 87.3 (17.8) |  |
| Median [min - max] | 88.9 [33.3 - 100.0] | 100.0 [44.4 - 100.0] |  |
|  |  |  |  |
| **Social contact** |  |  | **0,57** |
| Mean (SD) | 92.1 (12.6) | 93.7 (13.9) |  |
| Median [min - max] | 100.0 [60.0 - 100.0] | 100.0 [33.3 - 100.0] |  |
|  |  |  |  |
| **Sexuality** |  |  | **0,20** |
| Mean (SD) | 54.5 (42.9) | 75.0 (26.0) |  |
| Median [min - max] | 66.7 [0.0 - 100.0] | 66.7 [0.0 - 100.0] |  |
|  |  |  |  |
| **Teeth problems** |  |  | **0,02** |
| Mean (SD) | 48.5 (40.5) | 79.4 (35.8) |  |
| Median [min - max] | 33.3 [0.0 - 100.0] | 100.0 [0.0 - 100.0] |  |
|  |  |  |  |
| **Mouth problems** |  |  | **0,04** |
| Mean (SD) | 57.6 (36.8) | 80.4 (30.8) |  |
| Median [min - max] | 66.7 [0.0 - 100.0] | 100.0 [0.0 - 100.0] |  |
|  |  |  |  |
| **Dry mouth** |  |  | **0,21** |
| Mean (SD) | 27.3 (36.0) | 43.1 (38.1) |  |
| Median [min - max] | 0.0 [0.0 - 100.0] | 33.3 [0.0 - 100.0] |  |
| **Sticky saliva** |  |  | **0,05** |
| Mean (SD) | 30.3 (37.9) | 54.9 (34.7) |  |
| Median [min - max] | 33.3 [0.0 - 100.0] | 66.7 [0.0 - 100.0] |  |
|  |  |  |  |
| **Cough** |  |  | **0,18** |
| Mean (SD) | 66.7 (33.3) | 81.4 (23.5) |  |
| Median [min - max] | 66.7 [0.0 - 100.0] | 100.0 [33.3 - 100.0] |  |
|  |  |  |  |
| **Felt ill** |  |  | **1** |
| Mean (SD) | 93.9 (13.5) | 93.1 (16.0) |  |
| Median [min - max] | 100.0 [66.7 - 100.0] | 100.0 [33.3 - 100.0] |  |
|  |  |  |  |
| **Painkillers** |  |  | **0,73** |
| No | 8 (72.7%) | 22 (64.7%) |  |
| Yes | 3 (27.3%) | 12 (35.3%) |  |
|  |  |  |  |
| **Nutritional supplements** |  |  | **0,02** |
| No | 7 (63.6%) | 32 (94.1%) |  |
| Yes | 4 (36.4%) | 2 (5.9%) |  |
|  |  |  |  |
| **Lost weight** |  |  | **0,34** |
| No | 8 (72.7%) | 30 (88.2%) |  |
| Yes | 3 (27.3%) | 4 (11.8%) |  |
|  |  |  |  |
| **Gained weight** |  |  | **1** |
| No | 8 (72.7%) | 24 (70.6%) |  |
| Yes | 3 (27.3%) | 10 (29.4%) |  |
